# Case Report: Intramedullary Intervertebral Disk Extrusion in a Cat: Clinical, Computed Tomographic, High-Field Magnetic Resonance Imaging, and Outcome Findings

**DOI:** 10.3389/fvets.2020.583892

**Published:** 2020-10-07

**Authors:** Maud Debreuque, Isabelle Valin, Daniela Prata, Pauline De Fornel, Jean-Laurent Thibaud

**Affiliations:** ^1^MICEN VET, Créteil, France; ^2^Service de Médecine Interne, Université de Toulouse, ENVT, Toulouse, France; ^3^Clinique Michel Baron, Créteil, France; ^4^IDEXX Laboratoires, Saint-Denis, France

**Keywords:** peracute disk extrusion, intramedullary, high-field MRI, non-compressive, case report, CT, cat, Cauda Equina

## Abstract

**Background:** Intramedullary disk extrusions has rarely been described in veterinary medicine, more especially in cats, with only two cases are reported in the veterinary literature. Diagnosis may be difficult, even though clinical presentation and imaging studies, such as MRI or CT, can present specific features. Treatment and prognosis are not clearly described.

**Case presentation:** A 10-year-old domestic shorthair female cat was evaluated for a 12 h-history of peracute-onset of paraparesis with flaccid tail and urinary and fecal incontinence. The patellar reflexes were normal, the pelvic flexor reflexes were decreased (more markedly on the right limb) and the perianal reflex was absent. The tail was flaccid, without nociception. Abdominal palpation revealed a small urinary bladder, easily expressed. Manipulation of the lumbar vertebral column elicited marked pain. Neurological examination was consistent with a L7-caudal segments lesion. A lumbosacral MRI and CT evaluations were performed and revealed a focal intramedullary hemorrhagic lesion, with an associated vertical linear tract communicating with the L5-L6 intervertebral disk space, and a suspected intramedullary focus of mineralization. These imaging findings were highly suggestive of an L5-L6 intramedullary disk extrusion. A dorsal L5-L6 laminectomy confirmed the presence of intramedullary degenerative nucleus pulposus fragments, which were surgically removed. Rapid and progressive neurological improvement was observed post-surgery. At the 1-year follow-up, right plantigrade stance and mild paraparesis were still noticed, but jumps and voluntary tail movements were observed. Occasional urinary and fecal incontinence episodes remained.

**Conclusions:** This is the first feline case report of an intramedullary disk herniation with long-term follow-up available. Clinical description, CT and High-Field MRI findings, surgical procedure and histological results are reported, and help describing the characteristics of this rare non-compressive category of peracute intervertebral disk extrusion. Surgical management may be considered in feline cases of intramedullary disk herniation and may be associated with a good outcome.

## Background

Intervertebral disk disease rarely occurs in cats, representing ~5% of feline spinal cord disorders (1–3). Majority of cases concerns typical ventral extradural compression of degenerate extruded or protruded nucleus pulposus disk (1–3). However, with the increasing availability of cross-sectional imaging in veterinary medicine over the past years, additional types of intervertebral disk herniation have been more commonly identified in dogs and, to a lesser content, in cats (4–8), including intramedullary intervertebral disk extrusion. This type of intervertebral disk disease has a very rare occurrence and is characterized by the migration of extruded nucleus pulposus fragments within the spinal cord. Very few data has been published (9–14) about this neurologic condition, with only two feline cases reported in the literature (15, 16). Concerning those two cases, only a presumptive diagnosis was made based on the imaging results, without surgical or necropsy confirmation. In the present report, we describe the High-field MRI, CT features and long-term follow-up of a cat diagnosed with an intramedullary lumbar disk extrusion, confirmed and successfully treated surgically, and followed over 1 year.

## Case Presentation

A 10-year-old female spayed domestic shorthair cat was referred for peracute-onset of ambulatory paraparesis, more pronounced on the right, with a flaccid tail and fecal and urinary incontinence for the past 12 h. The cat was in the garden for a few hours and there were no known traumatic event reported. The patient had no history of spinal pain or neurological deficit prior to this episode. On physical examination, the cat was moderately overweight and the perineal hair were soiled with urine. The caudal abdominal palpation revealed a small urinary bladder, easily expressed. Neurological examination demonstrated normal thoracic limbs, ambulatory asymmetric paraparesis and mild pelvic ataxia, more pronounced on the right side. Pelvic postural reactions were delayed. Patellar reflexes and pain sensation were normal, and pelvic flexor reflexes were reduced, more markedly on the right side. Lumbosacral hyperalgesia was observed during palpation of vertebral column. The tail was flaccid without nociception. Anal tone and perineal reflex were absent. These neurologic signs were consistent with a cauda-equina syndrome with a neurolocalization caudal to the L6 spinal cord segment, with sciatic/L7, sacral and caudal spinal cord segments or nerve roots involvement.

**Figure 1:**
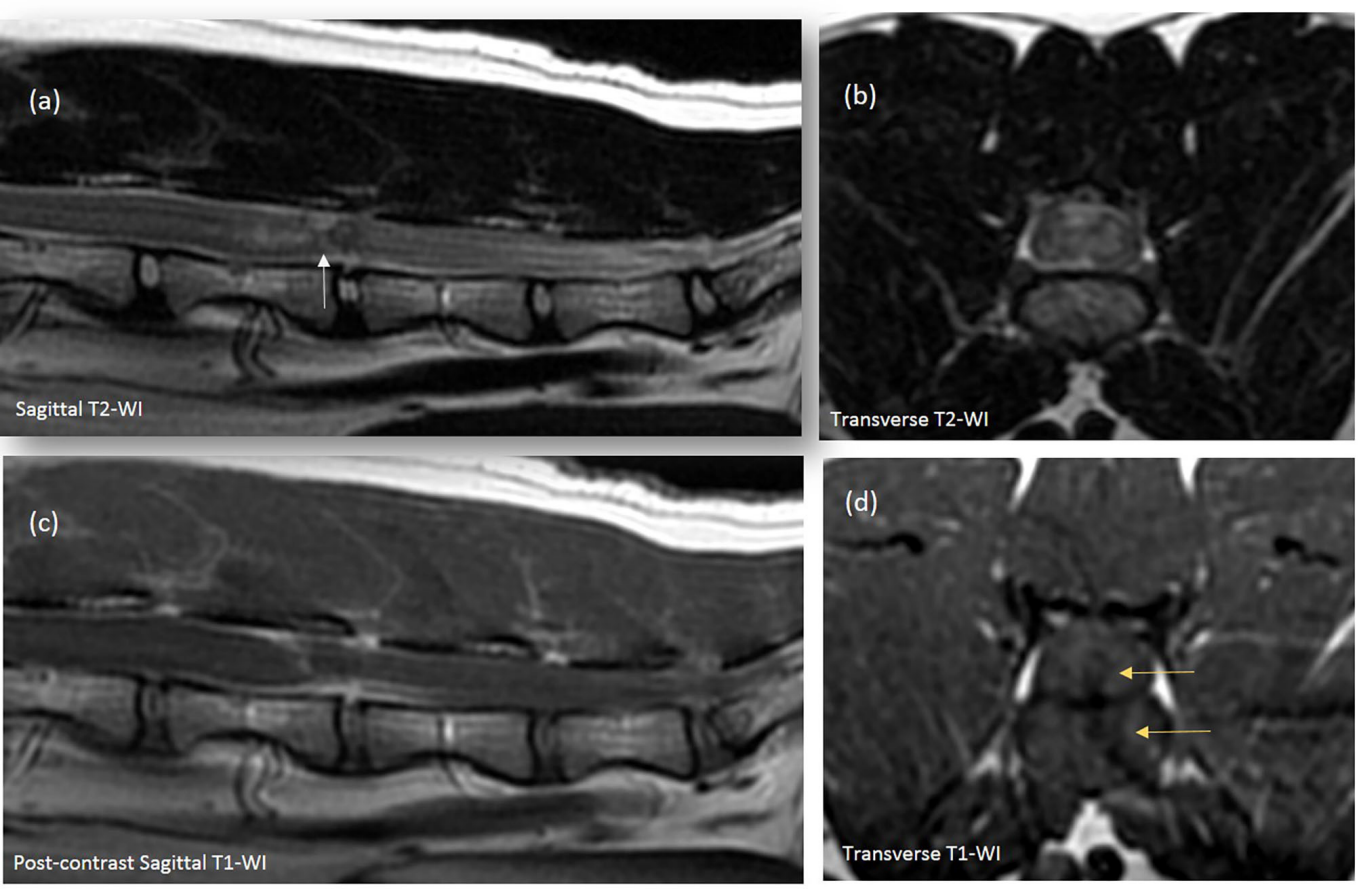
Sagittal **(a,c)** and transverse **(b,d)** images of the lumbar spinal cord. **(a,b)** There is a partial loss of the hyperintense signal of the L5-L6 IVD nucleus pulposus. **(a)** Overlying the L5-L6 IVD, a focal heterogenous spinal cord lesion, containing a linear hyperintense band, bordered by hypointense signal, extends from the dorsal aspect of the L5-L6 annulus fibrosus to the dorsal aspect of the spinal cord (white arrow). This vertical tract appears hypointense on the T1-WI transverse images [**(d)**, yellow arrows] and extends from the left aspect of the IVD space within the overlying spinal cord. Cranially, an ill-defined spinal cord hyperintense lesion and mild spinal swelling is observed, extending from mid L5 to L5-L6 IVD space **(a,b)**. **(c)** Mild contrast enhancement of the vertical intramedullary linear tract, meninges overlying L5-L6 IVD and adjacent to the area of spinal cord swelling can be observed a swell.

**Figure 2:**
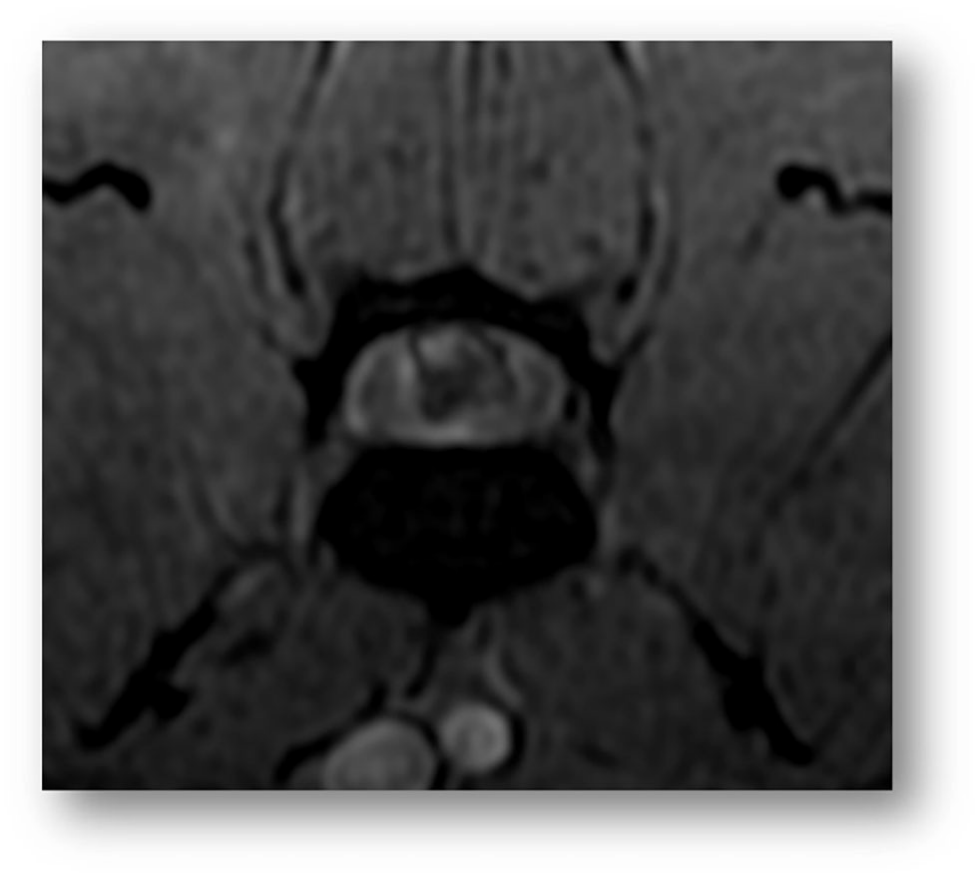
Transverse image of the lumbar spinal cord at the level of L5 (Gradient-echo sequence). A voluminous strongly hypointense rim surrounding a focal dorsal right intramedullary hyperintensity is observed, compatible with heterogeneous intramedullary hemorrhagic or mineralized lesion.

MRI of the lumbosacrocaudal region was performed using a high-field scanner^1^ (1.5-Tesla magnet). The MR images were acquired in a dorsal recumbency with a 3 mm slice thickness, and included transverse and sagittal T2-weighted images (T2-WI, TR 3000, TE 104 ms), T1-weighted images pre- and postcontrast (T1-WI and T1-WI+, TR 400, TE 9.2 ms) and transverse Multiple Echo Recombined Gradient Echo images (MERGE sequence, gradient echo sequence, TR 52.6, TE 18.3 ms). All intensities were compared to normal gray matter. On the sagittal T2-WI, there was partial reduction in volume of the L5-L6 nucleus pulposus (Figure 1a). Dorsal to this intervertebral disk (IVD) space, a focal relatively well-defined intramedullary heterogeneous lesion was observed on the T2-WI sequences (Figures 1a,b), partially correlated to a voluminous strongly hypointense rim surrounding a focal dorsal right-sided intramedullary hyperintensity on the transverse MERGE-WI (Figure 2). A vertical linear tract was also observed intralesionally, T2-WI hyperintense, T1-WI hypointense, extending within the spinal cord parenchyma from the dorsal L5-L6 annulus fibrosus (Figures 1a–d). Mild spinal cord swelling was also present, from the mid-vertebral body of L5 to the L5-L6 intervertebral disk space (Figure 1a).

**Figure 3:**
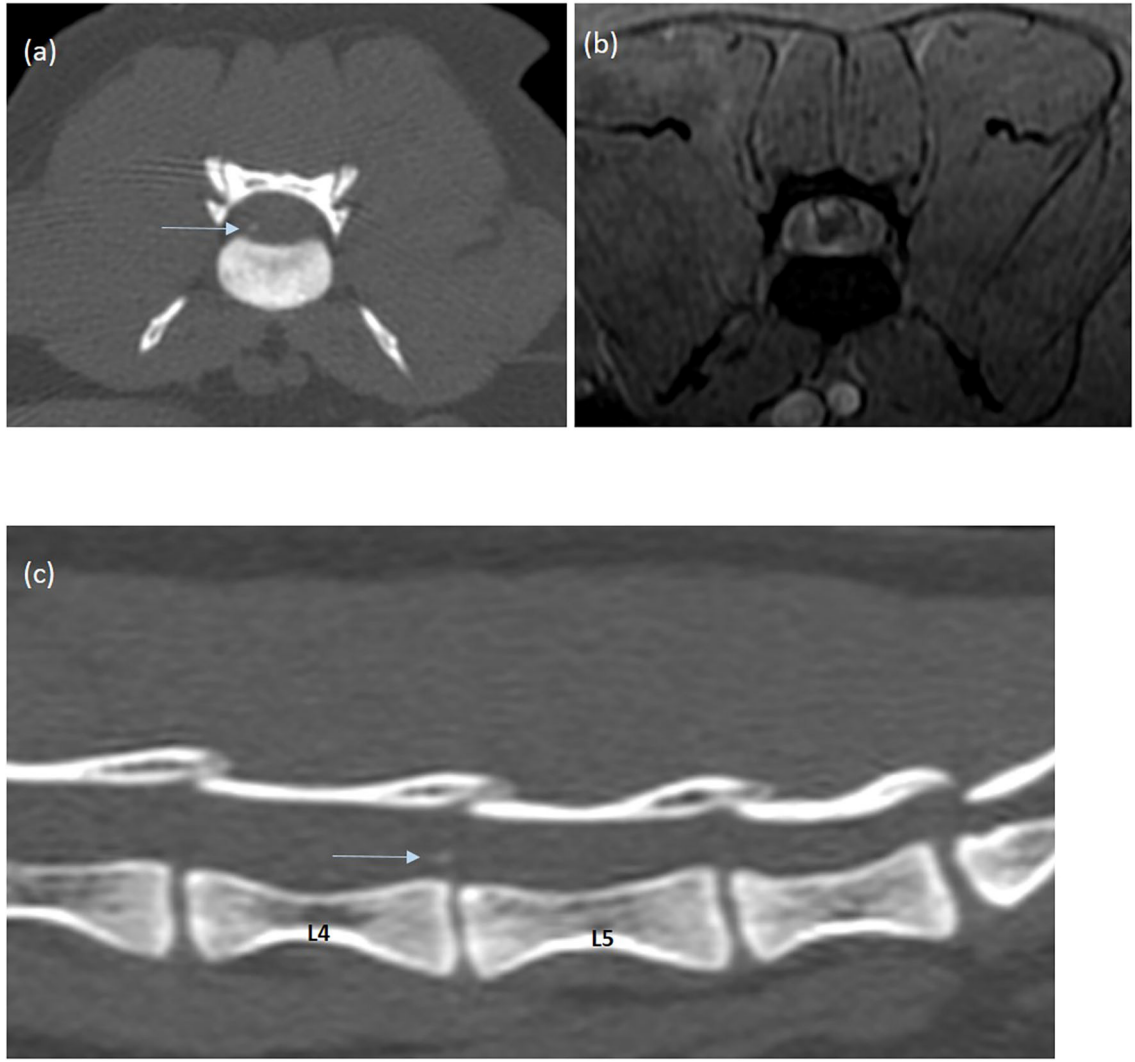
CT transverse **(a)** and sagittal **(c)** images of the lumbar spinal cord (acquired in a bone algorithm, and displayed in a bone window) and MRI gradient-echo transverse image **(b)** of the lumbar spinal cord. A right focal hyperattenuating intramedullary lesion with a CT number of 266 HU is observed at the dorsal aspect of the L5-L6 intervertebral space. The L5-L6 intervertebral disc space is partially collapsed compared to the adjacent intervertebral disk spaces.

After intravenous administration of Gadodiamide^2^ (0.05 mmol/mL, dose of 0.2 mL/kg), mild perilesional enhancement was noted around the vertical intramedullary linear tract, associated with focal L5-L6 meningeal enhancement (Figure 1c). Consideration for these MRI findings was given to an ischemic myelopathy with associated spinal cord edema and possibly intramedullary hemorrhage or focal mineralization. A lumbosacral Computed Tomography^3^ (CT) was performed to further assess the MRI findings. A faint hyperattenuating intramedullary focus was noted overlying the narrowed L5-L6 IVD space, most likely compatible with a focus of mineralization (266 Hounsfied Unit) (Figure 3). Based on the MRI and CT findings in this cat, primary consideration was given to intramedullary extrusion of mineralized nucleus pulposus material from the L5-L6 intervertebral disk, with suspected secondary intramedullary hemorrhage and edema.

**Figure 4:**
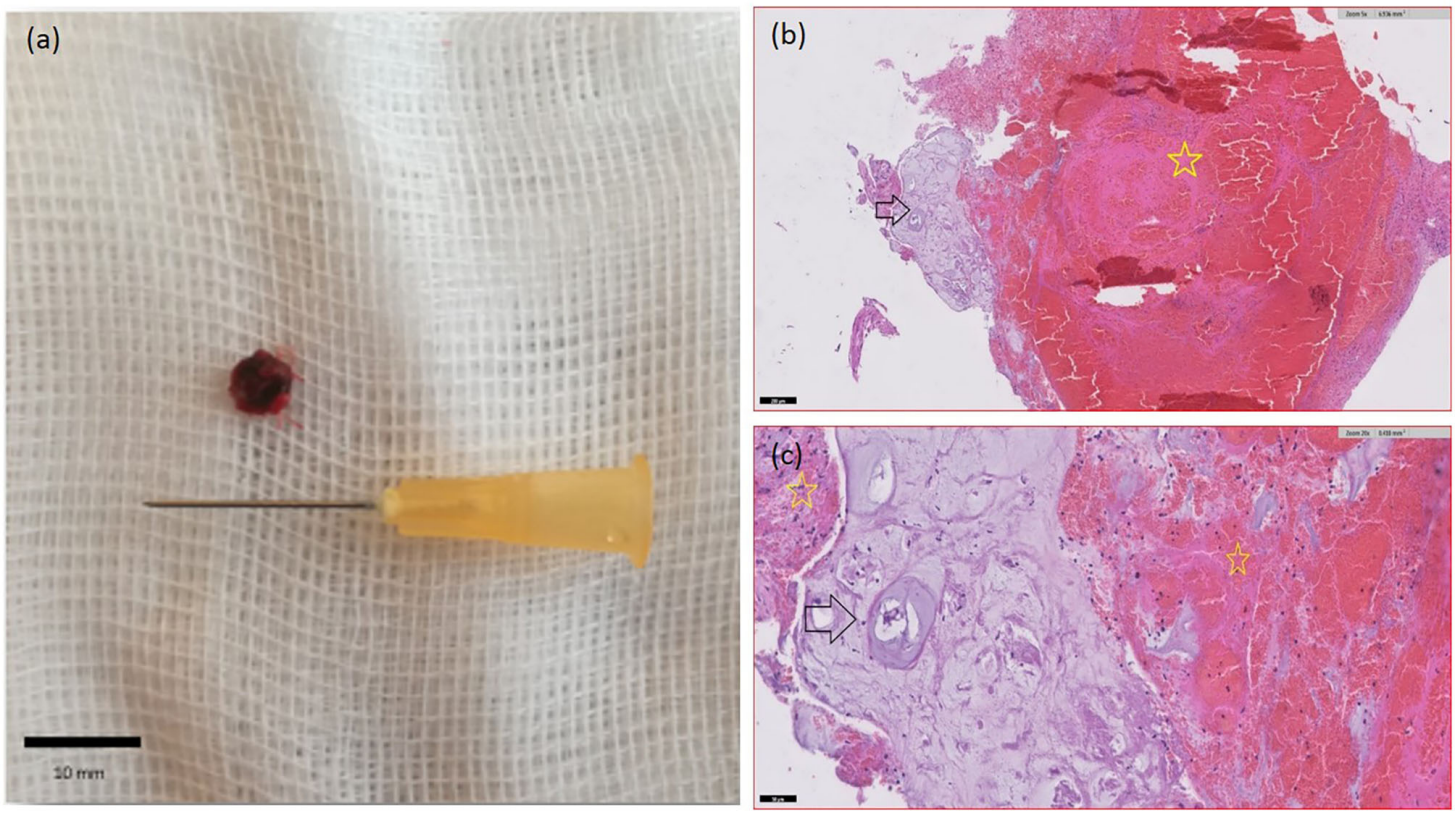
Intramedullary tissue surgically removed. A solid white material mixed with hematoma is observed. **(b,c)** Histological appearance of the material. Fragments of degenerate cartilaginous tissue (black arrows) consistent with cartilaginous metaplastic degeneration of IVD nucleus pulposus. Adjacent abondant fibrinohemorragic material (yellow stars). Haematoxylin and eosin stain. **(a)** x5. **(b)** x20.

A dorsal laminectomy was performed over the L5-L6 intervertebral space. The epaxial musculature was normal and there was no evidence of extruded disk material or hemorrhage within the dorsal extradural space. The dorsal dural tube was intact and enlarged, and a subdural mixed discolored and hemorrhagic focal spinal cord lesion was identified. A dorsal linear durotomy was performed, and the spinal cord appeared focally swollen with yellow-reddish color noted at this level. Parts of hematoma were gently removed and solid white material mixed with hemorrhage was observed (Figure 4a), within the spinal cord parenchyma, that appeared soft and discolored.

The histologic analysis on the surgically removed intramedullary material showed a fibrino-hemorrhagic material associated with fragments of degenerated cartilaginous tissue, independent of vascular structures, consistent with degenerated nucleus pulposus disk material (Figures 4b,c), which confirmed the imaging diagnosis of lumbar intramedullary mineralized intervertebral disk extrusion.

The lumbar pain of the cat improved shortly after the procedure. The urinary incontinence was assumed to be related to urinary bladder and striated urethral sphincter dysfunction secondary to neuronal lesion of the pelvic and pudendal nerves. Betanechol (parasympathomimetic agent, 2.5 mg q8 h, PO) and manual bladder expression were initiated to improve voiding, by stimulating bladder emptying and avoiding deleterious bladder distension. Voluntary micturitions remained rare 1-week postoperatively and manual bladder expression became more difficult, with moderate bladder distension, despite increased Betanechol dosage (5 mg q8 h, PO). Prazosin^4^ (alpha1-antagonist, 1 mg q12 h, PO) was prescribed to decrease smooth urethral sphincter resistance. As voluntary voiding improved, medical treatment was tapered and manual bladder voiding stopped overtime. Progressive neurological improvement of the plantigrade stance, paraparesis and tail movements were noticed over the past 2 months following surgery. One year after surgery, right plantigrade stance and mild paraparesis were still noticed, but voluntary tail movements were observed and the cat was again able to jump. Occasional fecal and urinary incontinence episodes (while sleeping) were still reported. Pelvic hopping reactions were improved and paw placement tests were normal. Left pelvic reflexes were intact but right pelvic flexor reflex remained decreased.

## Discussion and Conclusions

Intervertebral disk disease (IVDD) is uncommon in cats (1, 2) and mostly represented by compressive disk extrusion/protrusion with insidious or progressive course in animals aged over 8 years-old (1, 2, 17). The recent neuroimaging advances and increased MRI availability in veterinary medicine, have however permitted the diagnosis of more unusual types of feline acute IVDD, such as compressive hydrated nucleus pulposus extrusion (HNPE), acute non-compressive nucleus pulposus extrusion (ANNPE) and intradural/intramedullary intervertebral disk extrusion (5, 6).

As in our case, in the context of peracute onset of asymmetric myelopathy with minimal extradural involvement, three hypotheses should be prioritized: ischemic myelopathy (IM, mainly fibrocartilaginous embolic myelopathy), and peracute non-compressive disk extrusions (PNDE) represented by ANNPE or intramedullary intervertebral disk extrusion (IIVDE) (6, 8). Differentiation between IIVDE and IM or ANNPE is crucial for therapeutic decision, as surgery is not indicated in the two latter diseases (5–8). As in our study, the clinical presentation is usually similar between these three categories, with older cats being mostly affected, without apparent gender or breed predilection (4–6, 8). However, potential occurrence of the clinical signs in association with physical activity, and the presence of spinal hyperalgesia should orient toward PNDE (6). MRI may reveal a nonspecific edematous/ischemic spinal cord lesion in the three initial conditions, although the spinal cord abnormalities should be centered over an abnormal IVD space (narrowed with decrease T2-weighted hyperintensity of the nucleus pulposus) in PNDE cases (5, 6). Moreover, the presence of an intramedullary hemorrhagic pattern and a vertical linear tract extending from the IVD space within the spinal cord parenchyma, appears to be characteristic of IIVDE (4–7, 9). This tract may represent the intramedullary path of extruded nucleus pulposus disk material, with various degree of degeneration (10–13, 17), after perforation of the annulus fibrosus, dorsal longitudinal ligament and ventral meninges. In cases where myelography was performed, focal contrast medium migration into the spinal cord could be noted (12, 13), compatible with an intramedullary lesion communicating with the subarachnoid space. The CT findings in several cases of intradural/intramedullary IVD herniations have been described (12, 14) but none of them have demonstrated the presence of intradural/intramedullary mineralized fragments. In our cat, the CT images were compatible with extruded disk material presenting some degree of mineralization, but the histologic analysis did not confirm mineralization of the removed disk material. This could possibly be explained by incomplete removal of the extruded disk material, or by removal of the suspected mineralized fragment with the perispinal hemorrhage, or incapacity to access the mineralized fragment due to a dorsal surgical approach. Post-surgical imaging could have potentially helped for further evaluation, but was denied by the owner.

Considering previously published data on feline cases, IIVDE appear to more commonly originate from the caudal lumbar IVD spaces, while IM has been reported more frequently in the cervical spinal cord (6, 7, 18). The only two reported cases of feline IIVDE were at L4-L5 (16) and L5-L6 (17), similar to our patient. As in usual extradural IVD extrusion/protrusion, it has been hypothesized that the increase incidence of IVDD at the level of the caudal lumbar joints in cats may be attributable to the stance configuration and extreme range of motion of these intervertebral disk spaces in cats (19). Interestingly, in humans, more than 90% of intradural disk herniations are lumbar in origin, and the majority of them involves L4-L5 IVD space (20). The pathophysiology behind this finding is not fully understood in humans, but thinning of the ventral dura with age, and abnormally dense adhesions between the annulus fibrosus, the dorsal longitudinal ligament and the dura, are suspected mechanisms. These adhesions may result from chronic inflammatory processes, prior herniation or trauma. Although our patient had no history of previous lumbar spinal cord disease, the pathophysiology may be similar in feline cases of IIVDE as well, considering the similar signalment (older patients) and neurolocalization.

Due to the paucity of reported IIVDE cases, there is currently no treatment consensus or known prognosis available in veterinary medicine. In the seven canine cases published, four of the affected dogs underwent surgery, and half of them had a good outcome. For the two reported cats with IIVDE, one was euthanized at the time of diagnosis (17), and the second was medically managed (16). This last cat had shown paraplegia with loss on nociception on the right pelvic limb, and demonstrated partial motor function recovery, 6 days after the initial onset. However, no long-term follow up was available in this cat a definitive diagnosis of IIVDE was not achieved. In our case, surgical management was considered, due to the suspected presence of mineralized intramedullary disk material, which might have prevented spontaneous regression, maintaining a deleterious inflammatory and hemorrhagic environment, and elevated intraspinal pressure.

To the authors' knowledge, the prognostic significance of intramedullary hemorrhage for the recovery of dogs and cats with disk herniation has not been studied (21–23). However, in reports concerning typical extradural IVD herniation, the amount and extent of intramedullary and subdural hemorrhage seem to be significantly associated with the severity of the spinal cord injury at the site of disk extrusion, and myelomalacia extension (24). Intramedullary hemorrhage may contribute to increased intraspinal pressure (along with spinal cord edema and intramedullary disk fragments) and biochemical mechanisms, both participating to the extension of spinal cord destruction and eventually myelomalacia (24). We could potentially assume, as suspected in our case, that surgical removal of the intramedullary disk material and associated hematoma may help stabilizing the intraspinal pressure and consequently, limiting irreversible spinal cord lesion, preventing from spontaneous recovery. Our case might therefore suggest that surgical management could be prioritized over conservative medical treatment in cases of IIVDE. However, surgical risks, such as iatrogenic hemorrhage, have to be considered and further studies would be needed to confirm this assumption.

To the authors' knowledge, this is the first report of a confirmed feline case of intramedullary disk herniation with a successful outcome, after surgical treatment and long-term follow-up. It illustrates the clinical, CT and MRI findings contributing to the high suspicion of an unusual type of peracute intervertebral disk herniation in old animals. The presence of suspected intramedullary mineralized disk material associated with an hemorrhagic spinal cord lesion and vertical intramedullary linear tract overlying an IVD space, are highly suggestive of IIVDE. Because of the suspected mineralized feature of the extruded nucleus pulposus fragments, thus not easily degradable, and its consequences on the intraspinal environment, surgical excision might be recommended over conservative treatment. A favorable outcome, with rapid and progressive improvement might be expected, although irreversible neurologic deficits may persist.

## Data Availability Statement

The raw data supporting the conclusions of this article will be made available by the authors, without undue reservation.

## Ethics Statement

The authors declare that this work involved the use of client-owned animal only, and followed established international recognized high standards (Best practice) of individual veterinary clinical patient care. Therefore, ethical approval form a committee was not needed. Written informed consent was obtained from the owners for the participation of their animals in this study.

## Author Contributions

MD and J-LT were responsible for the case management and MRI studies. MD was responsible for writing the manuscript. PD was involved in the CT studies. IV performed the surgery. DP performed the histopathological evaluation. All authors read, corrected, and approved the final manuscript.

## Conflict of Interest

The authors declare that the research was conducted in the absence of any commercial or financial relationships that could be construed as a potential conflict of interest.

## Footnotes

^1^Signa HD23 optima 1,5T, General Electric Healthcare, Chicago, IL.

^2^Dotarem; Guerbet, France.

^3^16-row multidetector helical CT unit GE Healthcare.

^4^Minipress, Pfizer, France.

## Abbreviations

ANNPE: acute non-compressive nucleus pulposus extrusion
CT: computed tomography
HNPE: hydrated nucleus pulposus extrusion
IIVDE: intramedullary intervertebral disk extrusion
IM: ischemic myelopathy
IV: Intervertebral
IVDD: intervertebral disk disease
IVD: intervertebral disk
MERGE sequence: Multiple Echo Recombined Gradient Echo sequence (gradient echo sequence)
MRI: magnetic resonance imaging
PNDE: peracute non-compressive disk herniation
T2-WI: T2-weighted images
T1-WI: T1-weighted images
TE: Echo Time
TR: Repetition Time.
